# A novel ICK mutation causes ciliary disruption and lethal endocrine-cerebro-osteodysplasia syndrome

**DOI:** 10.1186/s13630-016-0029-1

**Published:** 2016-04-11

**Authors:** Machteld M. Oud, Carine Bonnard, Dorus A. Mans, Umut Altunoglu, Sumanty Tohari, Alvin Yu Jin Ng, Ascia Eskin, Hane Lee, C. Anthony Rupar, Nathalie P. de Wagenaar, Ka Man Wu, Piya Lahiry, Gregory J. Pazour, Stanley F. Nelson, Robert A. Hegele, Ronald Roepman, Hülya Kayserili, Byrappa Venkatesh, Victoria M. Siu, Bruno Reversade, Heleen H. Arts

**Affiliations:** Department of Human Genetics (855), Radboud Institute for Molecular Life Sciences, Radboud University Medical Centre, PO-Box 9101, 6500 HB Nijmegen, The Netherlands; Laboratory of Human Embryology & Genetics, Institute of Medical Biology, A*STAR, Singapore, Singapore; Medical Genetics Department, Istanbul Medical Faculty, Istanbul University, Istanbul, Turkey; Institute of Molecular and Cell Biology, A*STAR, Singapore, Singapore; Department of Human Genetics, David Geffen School of Medicine, University of California, Los Angeles, USA; Department of Pathology and Laboratory Medicine, David Geffen School of Medicine, University of California, Los Angeles, USA; Department of Biochemistry, University of Western Ontario, Room 4212A, 1151 Richmond Street N, N6A 5B7 London, ON Canada; Medical Genetics Program, London Health Sciences Centre, London, ON Canada; Children’s Health Research Institute, London, ON Canada; Department of Paediatrics, The Hospital for Sick Children, Toronto, ON Canada; Program in Molecular Medicine, University of Massachusetts Medical School, Worcester, MA USA; Robarts Research Institute, London, ON Canada; Medical Genetics Department, Koç University School of Medicine, Istanbul, Turkey

**Keywords:** Endocrine-cerebro-osteodysplasia syndrome, ECO, Intestinal cell kinase, ICK, Short-rib thoracic dysplasia syndrome, SRTD, Ciliopathy, Ciliary defects

## Abstract

**Background:**

Endocrine-cerebro-osteodysplasia (ECO) syndrome [MIM:612651] caused by a recessive mutation (p.R272Q) in Intestinal cell kinase (ICK) shows significant clinical overlap with ciliary disorders. Similarities are strongest between ECO syndrome, the Majewski and Mohr-Majewski short-rib thoracic dysplasia (SRTD) with polydactyly syndromes, and hydrolethalus syndrome. In this study, we present a novel homozygous *ICK* mutation in a fetus with ECO syndrome and compare the effect of this mutation with the previously reported ICK variant on ciliogenesis and cilium morphology.

**Results:**

Through homozygosity mapping and whole-exome sequencing, we identified a second variant (c.358G > T; p.G120C) in *ICK* in a Turkish fetus presenting with ECO syndrome. In vitro studies of wild-type and mutant mRFP-ICK (p.G120C and p.R272Q) revealed that, in contrast to the wild-type protein that localizes along the ciliary axoneme and/or is present in the ciliary base, mutant proteins rather enrich in the ciliary tip. In addition, immunocytochemistry revealed a decreased number of cilia in ICK p.R272Q-affected cells.

**Conclusions:**

Through identification of a novel *ICK* mutation, we confirm that disruption of *ICK* causes ECO syndrome, which clinically overlaps with the spectrum of ciliopathies. Expression of ICK-mutated proteins result in an abnormal ciliary localization compared to wild-type protein. Primary fibroblasts derived from an individual with ECO syndrome display ciliogenesis defects. In aggregate, our findings are consistent with recent reports that show that ICK regulates ciliary biology in vitro and in mice, confirming that ECO syndrome is a severe ciliopathy.

**Electronic supplementary material:**

The online version of this article (doi:10.1186/s13630-016-0029-1) contains supplementary material, which is available to authorized users.

## Background

We have previously described an extended consanguineous family from an Old Order Amish community affected with neonatal lethal endocrine-cerebro-osteodysplasia (ECO) syndrome, characterized primarily by endocrine, cerebral, and skeletal abnormalities [1]. This is the only family with ECO syndrome known to date. Through homozygosity mapping and candidate gene sequencing, we identified a homozygous missense mutation (c.815G > A [UCSC HG19: NM_014920] and p.R272Q [UCSC: NP_055735]) in *ICK*, which encodes the intestinal cell kinase [1]. The ICK protein belongs to the ros-cross hybridizing kinase (RCK) family, which consists of two other proteins, i.e., male germ cell-associated kinase (MAK) and MAPK/MAK/MRK overlapping kinase (MOK) [2–4]. Recent studies suggest that ICK is a ciliary protein, involved in ciliogenesis and regulation of intraflagellar transport (IFT) [5–8]. Here, we describe a new family with one affected fetus who was initially diagnosed with short-rib thoracic dysplasia (SRTD), a clinically and genetically heterogeneous group of disorders that are characterized by skeletal abnormalities, including a narrow thorax, short ribs, short and bowed tubular bones, and a number of extraskeletal features [MIM:PS208500]. Homozygosity mapping and whole-exome sequencing in the affected fetus revealed a novel homozygous missense mutation in *ICK* (c.358G > T; p.G120C), confirming that disruptions in this gene cause ECO syndrome, a disorder that shows marked clinical overlap with the short-rib thoracic dysplasia syndromes (SRTD), Majewski and Mohr-Majewski in particular [1]. Based on the clinical overlap between ECO syndrome and these other ciliopathies, and recent in vitro and mouse studies [5, 6] that showed that ICK is a ciliary protein, we tested whether either mutation (p.G120C or p.R272Q) affects cilium presence, morphology, and function in ciliated mouse Inner Medullary Collecting Duct 3 (mIMCD3) cells and in skin fibroblasts derived from a p.R272Q patient with ECO syndrome.

## Methods

### Collection of human blood samples and ethics consent and permissions

Blood samples were collected from twenty-two family members (I:1–I:4, II:1–II:15 and III:3–III:5) from family 1. Genomic DNAs (gDNA) were extracted from blood using a Qiagen kit (Cat# 74106) and from a fetal skin sample (III:6) by standard procedure. Parents gave their informed consent for study participation, and the study was approved by the Clinical Research Ethics Committees of Singapore (IRB #2013/1029/E) and Istanbul Medical Faculty with protocol number 2012.743-IRB2.1061.

### Collection of human fibroblasts lines and ethics statement

A fibroblast cell line from an Old Order Amish patient (family 2 in this paper) with ECO syndrome and two healthy controls from non-Amish (control I) and Amish (control II) communities were collected previously [1]. Tissues were obtained with informed consent, whereby it should be noted that parents provided informed consent for the patient with ECO syndrome. Material was collected with approval by the Office of Research Ethics of the University of Western Ontario with the following reference number: 07920E.

### Genotyping and homozygosity mapping

14 individuals, including I:2, I:4, II:1, II:4, II:8, II:9, II:10, II:11, II:12, II:13, III:3, III:4, III:5, and III:6, were genotyped using Illumina HumanCoreExome-12v1 BeadChips following manufacturer’s instructions. Call rates were above 99 %, and gender and relationship were verified using Illumina GenomeStudio software. Identical-By-Descent (IBD) mapping was performed by searching for homozygous regions in the unique affected individual using custom programs written in Mathematica (Wolfram Research, Inc.). Allowing 1 % error rate, all homozygous regions that were >2 cM were examined. Candidate regions were further refined by exclusion of common homozygous segments with any of the 13 unaffected family members.

### Whole-exome sequencing

One microgram of high-molecular weight gDNA extracted from fetus III:6 was used for exome capture with ION TargetSeq Exome Kit. DNA was sheared using Covaris M220 Focused-ultrasonicator (Covaris Inc., Woburn, MA, USA) to target an average fragment size of 200 bp. Shearing was followed by end repair, ligation of adapters, nick repair, purification, size selection and final amplification prior to exome capture as per TargetSeq protocol. The amplified DNA was cleaned with Ampure XP reagent (Agencourt, Boston, USA), and the DNA was eluted in 30 µl low TE buffer. The libraries were quantified using a Qubit 2.0 Fluorometer (Life Technologies, Carlsbad, CA, USA). The exome library was used for emulsion PCR on an Ion OneTouch System or Ion Chef System (Life Technologies, Carlsbad, CA, USA) following the manufacturer’s protocol. Each library was sequenced on an Ion Proton instrument (Life Technologies, Carlsbad, CA, USA) using one ION PI chip.

Sequence reads were aligned to the human reference genome (Human GRCh37 (HG19) build) using Torrent Mapping Alignment Program (TMAP) from the Torrent Suite (v4.2.1). PCR duplicates in the BAM file were identified by the Filter Duplicates plugin (v4.2) and removed. The variants were called using the Torrent Variant Caller (TVC) plugin (v4.2.1) and imported into Ion Reporter (v4.2), where each variant was annotated using the “annotate single sample variants” workflow. Information including the associated gene; variant location; quality score; coverage; amino acid change; predicted functional consequences using SIFT [9], PolyPhen2 [10], and Grantham [11]; phyloP conservation scores [12]; and 5000 genomes Minor Allele Frequencies were provided for each variant. Common SNPs referenced in the NCBI database (ftp://ftp.ncbi.nlm.nih.gov/pub/clinvar/vcf_GRCh37/) and the Exome Sequencing Project (http://evs.gs.washington.edu/EVS/) were filtered out. Variants that were predicted to be synonymous or not having a location on either a coding exon, UTR, splice site junction, or flanking intron were also removed. Variants were next compared to an in-house database of 138 previously sequenced samples. Those variants that were present in greater than 3 % of the previously sequenced samples were removed.

### Cloning

The ultimate ORF clone of human *ICK* cDNA (IOH38087) in pENTR221 [1] was used to transfer *ICK* into p733 (Life Technologies) through Gateway cloning (Life Technologies) according to the manufacturer’s instructions. The p733 vector encodes a monomeric Red Fluorescent Protein (mRFP). The p.G120C and p.R272Q mutations were introduced through site-directed mutagenesis, and constructs were validated with restriction digests and Sanger sequencing.

### Cell culture

Localization studies of mutant and wild-type mRFP-ICK were performed in two different cell lines, i.e., Human Embryonic Kidney Cells 293 with Tantigen of SV40 (HEK293T) and mouse inner medullary collecting duct 3 (mIMCD3) cells. HEK293T cells were cultured in Dulbecco’s modified eagle’s medium (DMEM) with 10 % fetal calf serum (FCS) and mIMCD3 cells in DMEM/F12 (1:1) supplemented with 10 % FCS. Fibroblasts from an Old Order Amish patient with ECO syndrome and two healthy controls from non-Amish (control I) and Amish (control II) were cultured for immunocytochemistry experimentation. Fibroblasts were cultured in DMEM supplemented with 20 % FCS. All cell lines were cultured at standard cell culture conditions.

### Transfections in HEK293T and mIMCD3 cells

To analyze the cellular localization of mRFP-ICK in HEK293T cells, these cells were cultured on sterile coverslips for 24 h. Cells were then transfected with p733 vector DNA encoding either wild-type or mutant (p.R272Q or p.G120C) mRFP-ICK using Lipofectamine (Life Technologies, Bleiswijk, The Netherlands) according to manufacturer’s instructions. 48 h post transfection, cells were fixed in ice-cold methanol for 10 min. After a quick wash with PBS, cells were permeabilized with 0.5 % Triton X-100 in PBS. A drop of DAPI containing Fluoromount-G (Southern Biotech, Birmingham, AL, USA) was added to the cells, and cover glasses were then placed on microscopic glass slides. The experiment was performed twice, and >35 cells were counted per condition. Microscopic analysis was conducted with a Zeiss Axio Imager Z2 fluorescence microscope (Zeiss, Sliedrecht, The Netherlands) equipped with an ApoTome slider. Images were obtained with ZEN 2012 software (Zeiss) and processed with Photoshop CS4 (Adobe Systems, San Jose, CA, USA) and FIJI software. The protocol to determine the ciliary localization of mRFP-ICK in mIMCD3 cells was largely similar compared to HEK293T studies; however, after culturing mIMCD3 cells for 24 h on sterile coverslips followed by 24 h of transfection, culture medium was replaced by serum-low medium containing 0.2 % FCS. mIMCD3 cells were grown in this medium for 24 h to induce ciliogenesis. Subsequently, immunocytochemistry and microscopy were performed. The experiment was performed in quadruplicate, and >50 cilia were analyzed per condition.

### Immunocytochemistry

The mutational effects of p.R272Q were analyzed in patient-derived fibroblasts from an affected individual from family 2 compared to fibroblasts from healthy non-Amish (control I) and Amish (control II) controls. The fibroblast cell lines were cultured on sterile coverslips for 24 hours. Subsequently, ciliogenesis was induced by replacing the culture medium for DMEM with 0.2 % FCS for 48 h, followed by immunostaining. For this purpose, cells were washed in PBS, fixed in 2 % paraformaldehyde in PBS for 20 min permeabilized in 1 % triton X-100 in PBS for 5 min. Prior to antibody binding, the cells were blocked for 30 min in 2 % bovine serum albumin (BSA) in PBS. The following primary antibodies were used: ARL13B (rabbit polyclonal, Proteintech Group, Manchester, United Kingdom, 1:500) and RPGRIP1L (custom-made guinea pig polyclonal; SNC039, 1:500). Primary antibodies were diluted in 2 % BSA/PBS and incubated for 1 h at room temperature. After washing with PBS, cells were incubated with fluorescently-labeled secondary antibodies for 30 min. These include anti-guinea pig IgG Alexa Fluor 568 and 647 (1:400), and anti-rabbit IgG Alexa Fluor 488 (1:400). These antibodies were all obtained from Life Technologies (Bleiswijk, The Netherlands). After washing cover glasses in PBS, a drop of DAPI containing Fluoromount-G (Southern Biotech, Birmingham, AL, USA) was added to the cells. Cover glasses were placed on microscopic glass slides, and microscopic analysis was performed as previously described.

## Results

### Clinical description of patient I

**Fig. 1:**
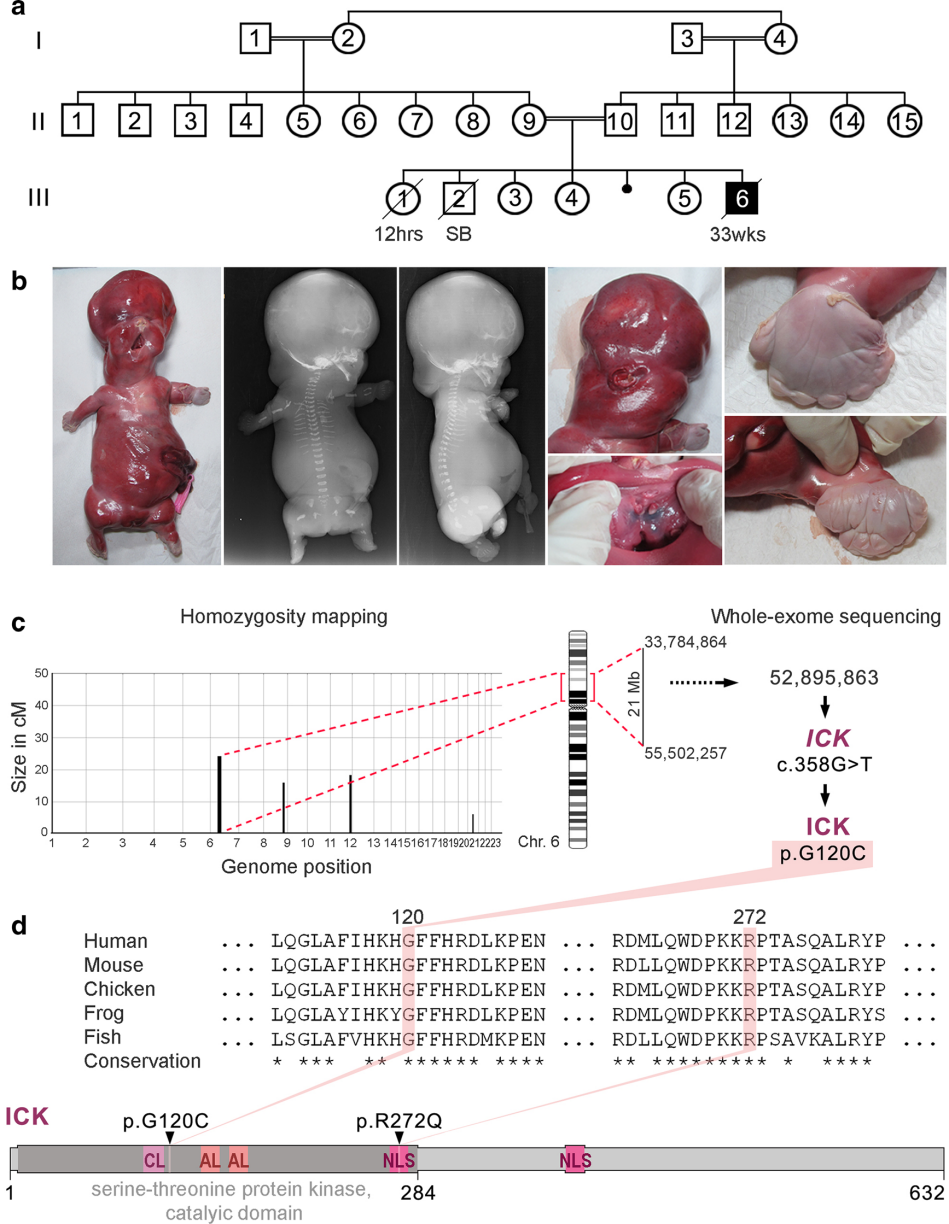
Phenotypic characteristics of a proband with endocrine-cerebro-osteodysplasia (ECO) syndrome caused by a new missense mutation in *ICK*. **a** Pedigree of Family 1. **b** Photographs and radiographs of the affected fetus. Note hydrops, tetramicromelia, narrow thorax, polydactyly with hypoplastic fingers and toes, and craniofacial anomalies including high forehead, deep set eyes, cleft lip and palate, natal teeth, multiple frenula of the upper lip, and low-set ears. **c** Homozygosity mapping delineated four candidate regions including one of 21 Mb on chromosome 6. Whole-exome sequencing of the fetus revealed a homozygous mismatch at position UCSC HG19 chr6: 52, 895, 863, causing one residue change p.G120C in ICK protein (NP_055735). **d** The mutated residue p.G120C is localized in the catalytic domain of the serine-threonine kinase ICK. The previously indentified variant p.R272Q that was found in ECO fetuses of an Old Order Amish family also resides in this domain. The glycine residue at p.120 is conserved in all vertebrate ICK homologs.* CL* catalytic loop;* AL* activation loop;* NLS* nuclear localization signal

The affected fetus from family 1 (III:6, patient I) was born to a consanguineous Turkish couple with three unaffected children (III:3, III:4, and III:5), a newborn who died 12 h after birth (III:1) due to an unknown etiology, and a still birth (SB) baby (III:2) (Fig. 1a). Antenatal ultrasound at 16 weeks of gestation showed cystic hygroma, scalp edema ascites, bilateral ventriculomegaly, very short tubular bones and polydactyly of hands and feet, and short ribs. A differential diagnosis of SRTD was made. Follow-up scan at 19 weeks of gestation revealed large and hyperechogenic kidneys, and a ventricular septal defect. Fetal karyotype was 46, XY. The pregnancy was terminated at 33 weeks of gestation. In addition to hydropic appearance, short thorax, and very short limbs, postmortem examination revealed oro-facial-digital defects with syndromic craniofacial features such as high and broad forehead, deep set eyes, hypertelorism, very small nose, anteverted nares, midline pseudocleft of the upper lip, clefting of the soft palate, natal teeth, labiogingival fusion, irregular alveolar crest, tongue fused to the lower palate, multiple frenula, micrognathia, low-set and posteriorly rotated ears, postaxial heptadactyly of the hands, and preaxial heptadactyly of the feet. Sex reversal with hypoplastic labial folds and anterior ectopic anus were also noted. Radiographs showed resemblance to SRTD type II, with very short tubular bones, torpedo-like femora, ovoid hypoplastic tibiae, premature epiphyseal ossifications in the lower limbs, short ribs, mildly defective ossification of the vertebrae, and small ilia with normal shape. Figure 1b shows various phenotypic characteristics of patient I. The clinical features observed in patient 1 significantly overlap with the features described of the affected infants in family 2, described by Lahiry et al. [1]. Table 1 shows a phenotypic comparison of the patients from both families.

**Table 1:** Clinical description and comparison of affected individuals carrying a homozygous mutation in *ICK*

Clinical features	Present fetus	Affected individuals combined/updated from Lahiry et al. [1]
	Pt I	IV-1, 2, 3, 4, 7, 8, new
Age at delivery (weeks)	33	Range 24–34
Karyotype	46, XY	46 (6/6), XY (4/6), XX (2/6)
Height (centile)	41 cm (10–25th)	3rd–10th (3/5), 90th–97th (2/5)
Weight (centile)	2450 g (90–97th)	90th–98th (5/7), 10th–25th (2/7)
Head circumference (centile)	34 cm (>97th)	90–>98th 4/6), 3rd–20th (2/6)
Autopsy	−	+ (3/6)
Oral		
Cleft palate	Cleft soft palate	Midline (5/6), notch in alveolar (1/6)
Cleft lip	Midline notching	Midline (5/7), bilateral (2/7)
Presence of premaxilla	+	+ (2/6)
Prominent upper lip region	−	+ (2/6)
Hypoplastic/absent epiglottis	Unknown	+ (2/3)
Hypoplastic/absent larynx	Unknown	+ (2/3)
Craniofacial		
Dolichocephaly	−	+ (3/6)
Midface hypoplasia	+	+ (6/6)
Microphthalmia	+	+ (4/6), cystic (2/6)
Retinal dysplasia	Unknown	+ (1/3)
Deep set eyes	+	+ (5/5)
Fused eyelids	−	+ (1/6)
Hypotelorism	−	+ (1/1)
Flat and wide nasal bridge	+	+ (6/6)
Dysplastic and low-set ears	+	+ (5/5)
Micrognathia	+	+ (6/6)
Excess skin below chin	−	+ (4/6)
Single nostril	−	+ (1/1)
Natal teeth	+	+ (1/6)
Limbs-thorax		
Micromelia	+, severe	+ (7/7)
Polydactyly (postaxial)	Upper limbs, heptadactyly	+ (6/6)
Polydactyly (preaxial)	Lower limbs, heptadactyly	−
Brachydactyly	+, Severe	+ (6/6)
Syndactyly	4 limbs, total syndactyly	+ (7/7)
Hitch-hikers’ thumbs	−	+ (1/6)
Palmar creases abnormalities	Irregular, vertical creases	+ (3/3)
Ulnar deviation of hands	−	+ (6/6)
Bowing of forearms	+	+ (6/6)
Bowing of lower legs	−	+ (2/6)
Abducted hips	+	+ (6/6)
Sandal gap	−	+ (6/6)
Talipes equinovarus	+	–
Chest width	Narrow	Broad (1/6), narrow (1/6)
Prominent xyphoid	−	+ (1/6)
Radiography		
Abnormal long bones (radius, ulna, tibia, fibula)	Very short with bowed radii and more severe involvement of the lower limb	Short diaphysis (1/1)
Short and incurved ulnae	+	+ (1/1)
Short and ovoid tibiae	Very short, with very short fibula	+ (1/1)
Abnormal humerus	Very short	Short diaphysis (1/1)
Abnormal femur	Very short, torpedo-like	Short and ovoid (1/1)
Spondylar involvement	+, Mildly defective ossification	Unknown
Premature epiphyseal ossification	+, Knees	Unknown
Abnormal ilium	Small ilia with normal contour	+ (1/1)
Abnormal/hypoplastic acetabular roof	−	+ (1/1)
Central nervous system		
Evidence of holoprosencephaly	−	+ (4/4)
Corpus callosum	+	+ (3/3)
Absence of septum pellucidum	+	+ (2/2)
Hydrocephalus (ventriculomegaly)	+	+ (2/2)
Dysmorphic cerebral aqueduct	Unknown	+ (2/2)
Olfactory bulbs	Unknown	+ (2/2)
Cerebral cortex malformation	Unknown	+ (3/3)
Brainstem malformation	Unknown	+ (3/3)
Cerebellar abnormalities	−	+ (2/2)
Hippocampus agenesis	Unknown	+ (2/2)
Leptomeningeal glioneuronal heterotopia	Unknown	+ (1/1)
Spinal cord malformation	Unknown	+ (1/2)
Endocrine system		
Pituitary gland	Unknown	+ Absent (2/2)
Adrenal glands	Unknown	+ Absent (1/3), hypoplastic (2/3)
Other		
Polyhydramnios	−	+ (1/6)
Fetal hydrops	+	−
Pulmonary hypoplasia	Unknown	+ (1/1)
Congenital heart defect	+, VSD	−
Gastrointestinal anomalies	Unknown	+ (2/3)
External genitalia abnormalities	Sex reversal with hypoplastic labial folds	+ (4/7)
Cryptorchidism	n/a	+ (1/6)^c^
(Hyper)echogenic kidneys	+	+ (3/6)
Large kidneys	+	+ (1/6)^d^
Squamous metaplasia of bladder	Unknown	+ (1/3)

Blank cells indicate that information was unavailable

^a^ Information gathered from attending-physician reports

^b^ Report from International Skeletal Dysplasia Registry (Cedars-Sinai Medical Center) for patient 1

^c^ Feature is reported as patient had ambiguous external genitalia

^d^ Large kidneys displayed cystic tubules

### Genotyping and homozygosity mapping

Homozygosity mapping identified IBD regions in the unique affected individual that were refined by exclusion of common homozygous segments with any of the 13 unaffected members of family 1. Four homozygous loci (>2 CM), totaling 50 Mb, were delineated on chromosomes chr6: 33, 784, 864–55, 502, 257; chr8: 119, 886, 091–129, 606, 949; chr11: 120, 548, 318–131, 461, 292; and chr20: 29, 834,107–37, 252, 342 (HG19) (Fig. 1c).

### Whole-exome sequencing

Whole-exome sequencing of the fetus III:6 gDNA generated 15.6 Gb of data with an average read length of 169 bp. An average coverage of 194X was achieved over the exome, with 96 % of bases covered at least 20X. A total of 34,881 variants were identified across protein-coding exons, UTRs, splice sites, and flanking introns. After applying all filters, a final set of 548 variants (49 homozygous and 499 heterozygous) were identified (Additional file 1: Table S1). Only 5 homozygous variants were found in the IBD blocks, of which 4 have been seen in population or affect a non-conserved amino acid, leaving only one new protein-changing variant (c.358G > T) in *ICK* (NM_014920.3) (Fig. 1c). Genotypes of the 376 flanking SNPs (Chr6: 50, 746, 213–54, 121, 367–HG19) were found homozygous only in the fetus compared to the 13 unaffected relatives (Additional file 2: Table S2). We also confirmed by Sanger sequencing that this *ICK* mutation was homozygous in the fetus, heterozygous in both parents and either heterozygous, or wild type in 20 other relatives (Additional file 3: Figure S1). To date (October 2015), this variant has not been reported in any public databases such as the online Exome Aggregation Consortium (ExAC) (http://exac.broadinstitute.org). Neither has the variant been reported in 1020 individuals who were whole exome sequenced by the Turkish National Genomics Research groups under TUBITAK. In addition, the p.G120C variant was not found in our in-house database containing exomes of 72 other Turkish individuals, which further underlines the rare character of this mutation. This missense mutation (p.G120C) leads to the alteration of a conserved glycine (Additional file 4: Figure S2) and is distinct from the ICK mutation p.R272Q previously reported in fetuses and newborns with ECO syndrome [1] (Fig. 1d). Both mutations are located in the serine-threonine protein kinase catalytic domain of ICK, while p.R272Q also affects a nuclear localization signal (Fig. 1d). To conclude, through identification of a second *ICK* mutation in a fetus who displays ciliopathy related features, we confirm that genetic changes in *ICK* gene cause ECO syndrome.

### Ciliary localization of ICK is altered in ICK mutants

**Fig. 2:**
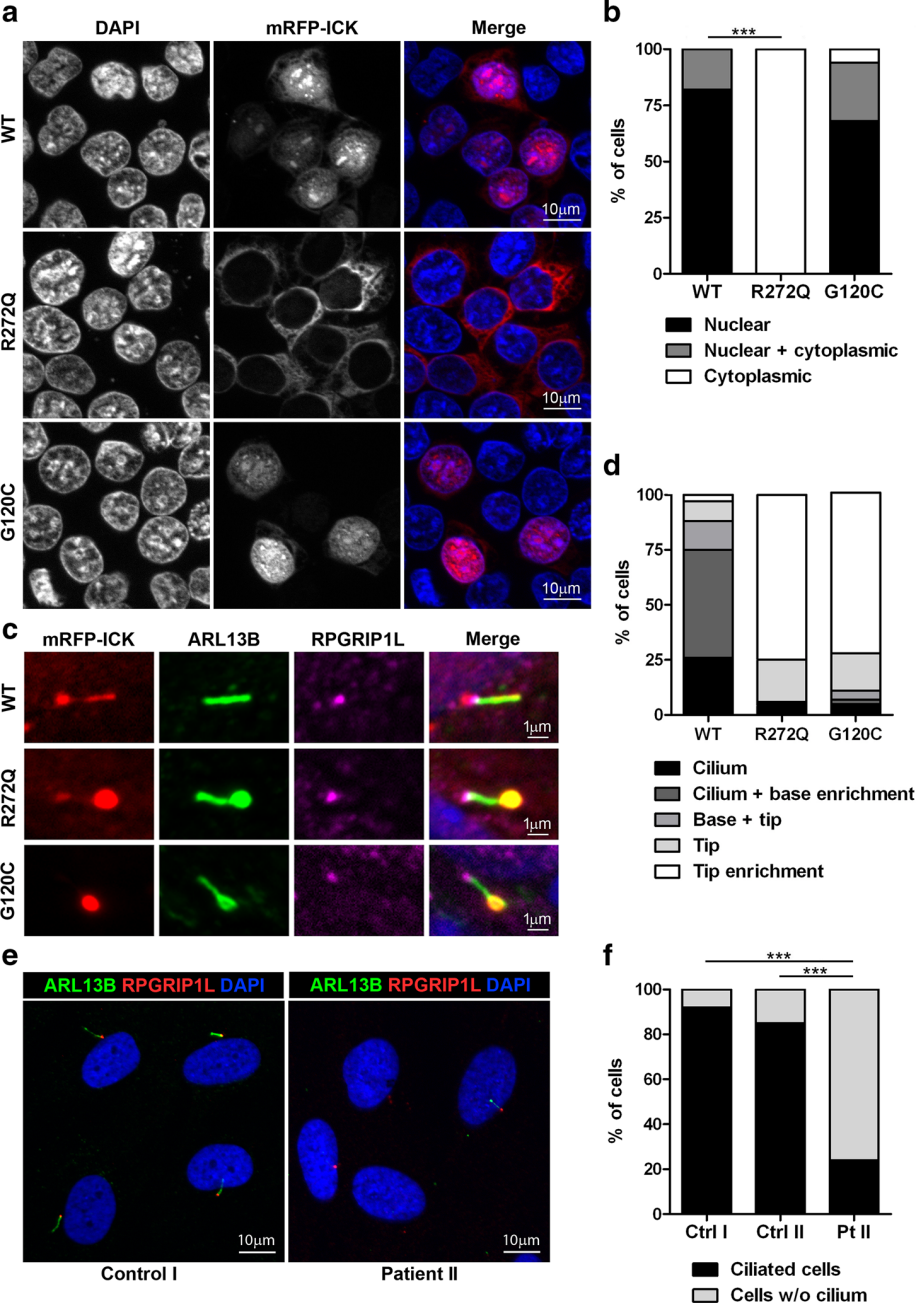
Mutations found in *ICK* affect ciliogenesis and ciliary localization. **a**, **b** The cellular localization of ICK was analyzed in HEK293T cells overexpressing wild-type or two mutated forms of mRFP-ICK: p.R272Q (positive control, mutation previously studied in ECO infants) and p.G120C (new mutation detected in the current study). Nuclei were stained with DAPI (blue) and mRFP-ICK proteins are shown in red. mRFP-ICK with the p.G120C mutation showed the same nuclear localization as wild-type mRFP-ICK transfected cells, while nuclear import was completely lost when mRFP-ICK with the p.R272Q mutation was expressed (Fisher’s exact test two-tailed *p* < 0.0001, >35 cells counted per condition). **c**, **d** The ciliary localization of ICK was studied in mIMCD3 cells transiently transfected with wild-type or two mutant forms of mRFP-ICK: p.R272Q or p.G120C. Wild-type mRFP-ICK mostly localized to the ciliary axoneme and was often enriched at the ciliary base, while both mRFP-ICK mutants enriched at ciliary tips. Ciliary axonemes were visualized with anti-ARL13B (*green*), ciliary transition zones that are present at the ciliary base were marked with anti-RPGRIP1L (*pink*), mRFP-ICK constructs were shown in *red*, and nuclei were stained with DAPI (*blue*). Per construct >50 transfected, ciliated cells were counted. **e**, **f** Cilium presence was studied in serum-starved fibroblasts derived from an ECO patient with a homozygous missense mutation in *ICK* (c.815G > A; p.R272Q) and compared to fibroblasts derived from two healthy unrelated controls. Control I represents a non-Amish individual, while control II is from the Amish community. Ciliogenesis was significantly reduced in fibroblasts from the ECO patient compared to the controls (Fisher’s exact test two-tailed *p* < 0.0001 for both). At least 80 cells were counted per condition. Ciliary axonemes were visualized with anti-ARL13B (*green*), ciliary transition zones were marked with anti-RPGRIP1L (*red*), and nuclei were stained with DAPI (*blue*)

We studied the cellular and ciliary localization of the two homozygous missense mutations in ICK, c.815G > A, p.R272Q known to cause ECO syndrome, and c.358G > T, p.G120C from the family reported here. The cellular localization of ICK was analyzed using HEK293T cells that were transiently transfected with mRFP-ICK constructs. As previously shown by Lahiry et al., wild-type ICK localized to the nucleus of these cells, whereas the ICK p.R272Q mutant remained in the cytoplasm. On the contrary, the novel ICK mutant p.G120C behaved as wild-type ICK protein and localized in the HEK293T cell nucleus (Fig. 2a, b). Since the mutation p.R272Q resides in the nuclear localization signal domain of ICK, it may affect the nuclear import of ICK protein. To examine the effect of p.R272Q and p.G120C mutations on ciliogenesis, we transiently transfected mIMCD3 cells with mRFP-ICK constructs and stimulated the cells to form cilia by serum starvation. While wild-type mRFP-ICK was often present in the ciliary axoneme with or without enrichment at the ciliary base (92 %), both ICK mutants were enriched at the ciliary tip in ~75 % of the cells (Fig. 2c, d). Figure S3 in Additional file 5 visualizes each of the ciliary phenotypes shown in Fig. 2d. Cilia of cells expressing mutant mRFP-ICK display an aberrant bulged tip in ~70 % of ciliated cells as visualized with ARL13B (Fig. 2c), while ciliary length is not essentially altered (Additional file 6: Figure S4). In summary, while the nuclear localization of ICK is only compromised by the p.R272Q mutation, we found that the ciliary localization is affected by both p.R272Q and p.G120C mutants.

### Ciliogenesis is reduced in ECO patient-derived fibroblasts

To study the endogenous effect of the homozygous missense mutation p.R272Q on cilia, we monitored ciliogenesis and ciliary length in primary fibroblasts derived from an ECO patient (Patient II) and compared them to cells derived from two healthy controls, one non-Amish (Control I) and the other from the Amish community (Control II). While 92 and 85 % of the control cells were ciliated, only 24 % of patient-derived cells formed cilia (Fig. 2e, f). This was determined with a Fisher’s exact two-tailed test that showed *p* < 0.0001 when comparing the patient sample to each of the controls. We did not observe any major differences in ciliary length (Additional file 7: Figure S5). In summary, we found that ECO patient-derived fibroblasts display reduced ciliogenesis, while the cilia that form are of normal length.

## Discussion

In this study, we identified a novel homozygous missense mutation (c.358G > T [UCSC HG19: NM_014920] and p.G120C [UCSC: NP_055735]) in the *ICK* gene in a consanguineous Turkish family with one affected fetus who was initially clinically diagnosed as SRTD type II. After mutation identification, the clinical features of the proband were compared to those of the affected progeny of a previously published Amish family with ECO syndrome who had a different homozygous missense mutation in ICK (p.R272Q) [1]. Although it remains unclear if the Turkish proband had typical endocrine abnormalities seen in ECO syndrome, there was significant clinical overlap with Amish fetuses, which leads us to conclude that we identified the second family with ECO syndrome world-wide. This study thus confirms that aberrations of *ICK* can indeed cause severe phenotypes that resemble the short-rib thoracic dysplasia spectrum. Our in vitro studies performed in mIMCD3 cells show that mutant mRFP-ICK with either p.R272Q or p.G120C accumulates in the ciliary tip, while the wild-type protein localizes along the ciliary axoneme with enrichment in the ciliary base. Our findings are in line with the data published by Moon et al. [6] who reported similar localization patterns for both wild-type and mutant ICK (p.R272Q). In contrast, Chaya et al. [5] showed that wild-type ICK localizes to the ciliary tip and that this localization is lost in the p.R272Q mutant. These different observations in ICK localization may be due to variation in the expression level of ICK as well as the type and the position of tags in the protein. Typical accumulations of protein at ciliary tips were also observed in fibroblasts derived from patients with ciliopathies such as asphyxiating thoracic dystrophy [MIM:208500] [13] and cranioectodermal dysplasia [MIM:218330] [14]; cilia of these patients often display abnormal ciliary tip accumulation of intraflagellar transport complex B (IFT-B) proteins. The protein accumulations at ciliary tips may be due to defective IFT and disruption in retrograde intraflagellar transport, i.e., transport occurring from the ciliary tip to its base [14]. Thus, the protein accumulations at the ciliary tip that we observe may also be caused by defective IFT and suggest a role for ICK in the regulation of this process. Recent papers by Broekhuis et al. and Chaya et al. [5, 8] indeed indicate that ICK coordinates IFT probably through phosphorylation of KIF3A, a motor protein that drives IFT from the ciliary base towards the tip. Considering the presence of p.G120C and p.R272Q mutations in the serine-threonine protein kinase domain, it is tempting to speculate that both mutations affect phosphorylation of KIF3A, thereby dysregulating IFT. While various reports suggest that ICK is a regulator of cilia length [5, 6, 8], we did not observe dramatic differences in the length of cilia expressing mutant ICK nor in those cilia occurring on the apical surface of cells derived from an ECO patient (Additional file 6: Figure S4 and Additional file 7: Figure S5). This may be in part be explained by the likely hypomorph effects of the ECO syndrome-associated missense mutations. We did, however, observe a significantly reduced number of cilia in ECO patient-derived fibroblasts compared to two healthy controls. This may explain the previously described hyperproliferative behavior of these cells [15]. Our reasoning is that cilium formation depends on docking of the mother centriole of the centrosome to the plasma membrane from which the cilium can then emerge. When cilia are unstable or do not form, such as in ECO patient-derived cells, centrioles become available for assembly of the mitotic spindle promoting cell division. The ciliogenesis defect seen in ECO patient-derived fibroblasts is comparable to the defects observed in *Ick*-deficient mouse embryonic fibroblasts (MEFs), recently described by Chaya et al. [5].

Loss of *Ick* results in aberrant ciliary Hedgehog signaling in multiple murine organs and fibroblasts [5, 6], and this pathway is also dysregulated in other ciliary chondrodysplasias that share clinical features with ECO syndrome and SRTD [16–19]. It is currently unclear how the Hedgehog pathway is affected upon *Ick* depletion. One hypothesis is that loss of *Ick* directly affects IFT thereby altering Hedgehog signaling [5]. Another hypothesis is that *Ick* depletion impacts mTORC1 signaling thereby causing an imbalance in ciliary tubulin synthesis resulting in structural ciliary defects that in turn impact ciliary signaling [8]. In any case, many ECO and SRTD syndrome-associated anomalies are probably due to disturbances of ciliary signaling cascades regulating human development, including but probably not limited to the Hedgehog pathway.

## Conclusions

In conclusion, we here report a novel *ICK* mutation in a family with ECO syndrome. This is the first replicative allele, which confirms that *ICK* mutations are indeed associated with severe SRTD resembling phenotypes. We found ciliogenesis defects in fibroblasts derived from a previously reported individual affected by ECO syndrome and noted that expression of mutant ICK (p.R272Q and p.G120C) results in aberrant localization of ICK in ciliary tips. Although ciliary phenotypes vary slightly between cells and species, our findings are in agreement with recent reports on *Ick* knockout mice and murine *Ick* depleted cells that reveal a role for Ick in ciliogenesis, ciliary transport, and regulation of cell signaling. Our study is the first to show that ciliary defects occur in cells derived from a patient with ECO syndrome with mutated *ICK* and thereby provides additional support for inclusion of ECO syndrome in the severe ciliary disease spectrum.

## Additional files

10.1186/s13630-016-0029-1 Filtering of WES data.
10.1186/s13630-016-0029-1 SPN genotypes flanking the p.G120C mutation present in the fetus and 13 unaffected relatives.
10.1186/s13630-016-0029-1 The ICK missense mutation (c.358G > T; p.G120C) segregates with disease.
10.1186/s13630-016-0029-1 Alignment of the protein kinase domain in ICK, MAK and MOK in different species.
10.1186/s13630-016-0029-1 Different classes of ciliary phenotypes.
10.1186/s13630-016-0029-1 Ciliary length is not essentially altered in cilia of cells expressing mutant mRFP-ICK.
10.1186/s13630-016-0029-1 Ciliary length is not markedly altered in ECO patient-derived fibroblasts.

## Abbreviations

ECO: endocrine-cerebro-osteodysplasia
ExAC: Exome Aggregation Consortium
IBD: identical-by-descent
ICK: intestinal cell kinase
IFT: intraflagellar transport
IFT-B: intraflagellar transport complex B
MAK: male germ cell-associated kinase
MOK: MAPK/MAK/MRK overlapping kinase
MEFs: mouse embryonic fibroblasts
MIM: Mendelian inheritance in man
mTORC1: mammalian target of rapamycin complex 1
RCK: ros-cross hybridizing kinase
SRTD: short-rib thoracic dysplasia
UCSC: UCSC Genome Browser
