# Supplementary material for: A novel ICK mutation causes ciliary disruption and lethal endocrine-cerebro-osteodysplasia syndrome
Source: Cilia. 2016 Apr 11;5:8. doi: 10.1186/s13630-016-0029-1 (PMC4827216; doi:10.1186/s13630-016-0029-1)

Additional file 3: Figure S1. The ICK missense mutation (c.358G>T; p.G120C) segregates with disease. Sanger sequencing was performed on the proband and 22 healthy relatives.

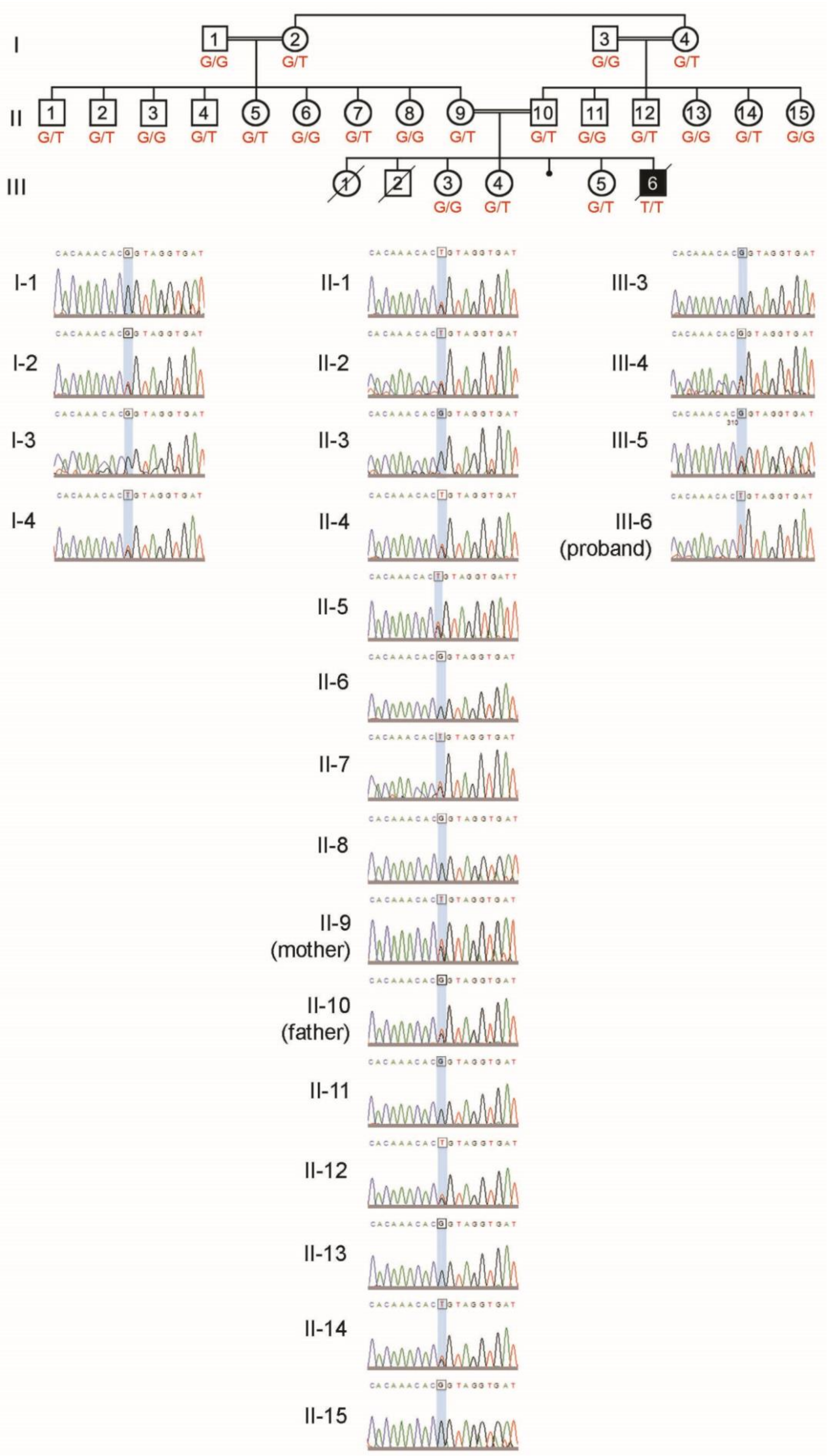

Supplement: Supplementary file 3 — 10.1186/s13630-016-0029-1 The ICK missense mutation (c.358G > T; p.G120C) segregates with disease. [file 13630_2016_29_MOESM3_ESM.pdf]
