# Supplementary material for: A novel ICK mutation causes ciliary disruption and lethal endocrine-cerebro-osteodysplasia syndrome
Source: Cilia. 2016 Apr 11;5:8. doi: 10.1186/s13630-016-0029-1 (PMC4827216; doi:10.1186/s13630-016-0029-1)
Supplement: Supplementary file 4 — 10.1186/s13630-016-0029-1 Alignment of the protein kinase domain in ICK, MAK and MOK in different species. [file 13630_2016_29_MOESM4_ESM.pdf]

Additional file 4: Figure S2. Alignment of the protein kinase domain in ICK, MAK and MOK in different species. The protein kinase domain in ICK, MAK and MOK is a conserved region, and it includes both described mutations in ICK, i.e. p.G120C and p.R272Q. The alignment is based on sequences from proteins with the following UniProt identifiers; Q9UPZ9 (*H. sapiens* ICK), Q9JKV2 (*M. musculus* ICK), B3WFY8 (*C. elegans* DYF-5b), P20794 (*H.sapiens* MAK), Q04859 (*M. musculus* MAK), Q7ZTX0 (*D. rerio* MAK), Q9UQ07 (*H. sapiens* MOK), Q9WVS4 (*M. musculus* MOK), Q9VL64 (*D. melanogaster* CG42366). The alignment was made using the online tool Clustal omega 1.2.1: http://www.ebi.ac.uk/Tools/msa/clustalo/.The annotations made by this tool visualize the conservation of each residue; an asterisk indicates "positions which have a single, fully conserved residue", a colon and a period indicate "conservation between groups of strongly similar properties" and "weakly similar properties", respectively. The colors assigned by Clustal omega inform about the properties of individual amino acids; red indicates small residues, blue indicates acidic residues, magenta shows basic residues, and green refers to amino acids with hydroxyl, sulfydryl and amine groups or to glycine residues. The protein sequences for each of the presented species was obtained from UniProt (www.uniprot.org). TreeFam (www.treefam.org), and ZFIN (http://zfin.org) websites were used to identify proteins that are homologues to ICK.

```
H.sapiens ICK           YTTIRQLGDGTYGSVLLGRSIESGELIAIKKMKRKFYSWEECMNLREVKSLKKLN-HANV   59
M.musculus ICK          YTTIKQLGDGTYGSVLLGRSIESGELIAIKKMKRKFYSWEECMNLREVKSLKKLN-HANI   59
C.elegans DYF-5b        YLMTKRLGDGTFGEVMLAKKIDTGDRVAIKRMKKKFYSWEEAMSLREVKSLKKLN-HPNI   59
H.sapiens MAK           YTTMRQLGDGTYGSVLMGKSNESGELVAIKRMKRKFYSWDECMNLREVKSLKKLN-HANV   59
M.musculus MAK          YTTMKQLGDGTYGSVLMGKSNESGELVAIKRMKRKFYSWDECMNLREVKSLKKLN-HANV   59
D.rerio MAK             YTTLKQLGDGTYGSVLMGKSNESGELVAIKRMKRKFYSWEECMNLREVKSLKKLN-HANV   59
H.sapiens MOK           YKAIGKIGEGTFSEVMKMQSLRDGNYYACKQMKQRFESIEQVNNLREIQALRRLNPHPNI   60
M.musculus MOK          YKAIGKIGEGTFSEVMKMQSLRDGNYYACKQMKQHFESIEQVNSLREIQALRRLNPHPNI   60
D.melanogaster CG42366  YITLTQLGDGTYGTVVLGQRKDTGEKVAIKRMKRKYYSWEEAMNLREVKSLKKLS-HPNI   59
                                       :       *:  * * ::            **::     . * *:

H.sapiens ICK           VKLKEVI-----RENDHLYFIFEYMK-ENLYQLIKER-NKLFPESAIRNIMYQILQGLAF   112
M.musculus ICK          VKLKEVI-----RENDHLYFIFEYMK-ENLYQLIKER-NKLFPESAIRNIMYQILQGLAF   112
C.elegans DYF-5b        IKLREVI-----RENDILYFVFEFMQ-ENLYELMKDRD-RYFPESVIRNIIYQVLQGLAF   112
H.sapiens MAK           IKLKEVI-----RENDHLYFIFEYMK-ENLYQLMKDR-NKLFPESVIRNIMYQILQGLAF   112
M.musculus MAK          IKLKEVI-----RENDHLYFVFEYMK-ENLYQLMKDR-NKLFPESVIRNIMYQILQGLAF   112
D.rerio MAK             VKLKEVI-----RENDHLYFVFEYMK-ENLYQLMKDRENKMFTENEIRNIMFQVLSGLAF   113
H.sapiens MOK           LMLHEVV---FDRKSGSLALICELMD-MNIYELIRGRR-YPLSEKKIMHYMYQLCKSLDH   115
M.musculus MOK          LALHEVV---FDRKSGSLALICELMD-MNIYELIRGRR-HPLSEKKIMLYMYQLCKSLDH   115
D.melanogaster CG42366  VKLKEVI-----RENDTLYFVFEYMK-ENLYQMIKDRD-THLPEPELKSILFQVLTGLAF   112
                        : : ::      . .  * :: * :.  :: . :. :      *  :   : *:  .: .

                          G120C
                            |
H.sapiens ICK           IHKHGFFHRDLKPENLLCMGPE---LVKIADFGLAREIRSKPPYTDYVSTRWYRAPEVLL   169
M.musculus ICK          IHKHGFFHRDLKPENLLCMGPE---LVKIADFGLAREIRSRPPYTDYVSTRWYRAPEVLL   169
C.elegans DYF-5b        MHKNGFFHRDMKPENIMCNGTE---LVKIADFGLAREIRSKPPYTDYVSTRWYRAPEILL   169
H.sapiens MAK           IHKHGFFHRDMKPENLLCMGPE---LVKIADFGLARELRSQPPYTDYVSTRWYRAPEVLL   169
M.musculus MAK          IHKHGFFHRDMKPENLLCMGPE---LVKIADFGLARELRSQPPYTDYVSTRWYRAPEVLL   169
D.rerio MAK             VHKHGFFHRDMKPENLLCMGPE---LVKIADFGLAREIRSRPPYTDYVSTRWYRAPEVLL   170
H.sapiens MOK           IHRNGIFHRDVKPENILIK-QD---VLKLGDFGSCRSVYSKQPYTEYISTRWYRAPECLL   171
M.musculus MOK          MHRNGIFHRDVKPENILVK-QD---VLKLGDFGSCRSVYSKQPYTEYISTRWYRAPECLL   171
D.melanogaster CG42366  MHRHGFFHRDLKPENLLCSGPD---LIKIADFGLAREIRSRPPFTDYVSTRWYRAPEVLL   169
                        :*   : ***:* **::        : *:.*:* .:    .    .  .* :*  ** *

H.sapiens ICK           RSTNYSSPIDVWAVGCIMAEVYTLRPLFPGASE-I--DTIFKICQVLGTPKKTDWPEGYQ   226
M.musculus ICK          RSTNYSSPIDIWAVGCIMAEVYTLRPLFPGASE-I--DTIFKICQVLGTPKKTDWPEGYQ   226
C.elegans DYF-5b        RSTSYNSPIDMWALGCIMAELYILRPLFPGTSE-M--DQLFKIISILGTPNKDEWPEGYQ   226
H.sapiens MAK           RSSVYSSPIDVWAVGSIMAELYMLRPLFPGTSE-V--DEIFKICQVLGTPKKSDWPEGYQ   226
M.musculus MAK          RSSVYSSPIDVWAVGSIMAELYTFRPLFPGTSE-V--DEIFKICQVLGTPKKSDWPEGYQ   226
D.rerio MAK             RSPVYSSPIDIWAVGCIMAELYTLRPLFPGNSE-V--DEIFKICQVLGTVKKSDWPEGHQ   227
H.sapiens MOK           TDGFYTYKMDLWSAGCVFYEIASLQPLFPGVNE-L--DQISKIHDVIGTPAQKIL-TKFK   227
M.musculus MOK          TDGFYTYKMDLWSAGCVFYEIASLQPLFPGVNE-L--DQISKIHDVIGTPCQKTL-TKFK   227
D.melanogaster CG42366  HSTNYGSTIDLWAMGCIMAELYTFRPLFPGSSE-V--DQLFKICSVLGTPDKDDWPDGYR   226
                            *    *:*: *  :  :    * * . .  :      :* .   :  :

                                                        R272Q
                                                          |
H.sapiens ICK           LSSAMNFRWPQCVPNNLKTLIPNASSEAVQLLRDMLQWDPKKRPTASQALRYPYF   281
M.musculus ICK          LSSAMNFLWPQCIPNNLKTLIPNASSEAIQLLRDLLQWDPKKRPTASQALRYPYF   281
C.elegans DYF-5b        LASAMNFRFQQVVATPMEQVVNTISKEGMKLMMDMMLWNPEKRPNANQSLRYKYF   281
H.sapiens MAK           LASSMNFRFPQCVPINLKTLIPNASNEAIQLMTEMLNWDPKKRPTASQALKHPYF   281
M.musculus MAK          LASSMNFRFPQCIPINLKTLIPNASSEAIQLMTEMLNWDPKKRPTASQALKHPYF   281
D.rerio MAK             LASAMNFRFPQCVPTPLKTLIPNATNEALDIMRDLLQWDPKKRPSAVKALRYPYF   282
H.sapiens MOK           QSRAMNFDFPFKKGSGIPLLTTNLSPQCLSLLHAMVAYDPDERIAAHQALQHPYF   282
M.musculus MOK          QSRAMSFDFPFKKGSGIPLLTANLSPQCLSLLHAMVAYDPDERIAAHQALQHPYF   282
D.melanogaster CG42366  LASMIHFRYPDCIKVPLSSVVSRCSQNGLDLLEDMLAYDPDKRPTAQQSLKYPYF   281
                                                : :  . :  ::  :*..*    : :
```
