# Supplementary material for: A novel ICK mutation causes ciliary disruption and lethal endocrine-cerebro-osteodysplasia syndrome
Source: Cilia. 2016 Apr 11;5:8. doi: 10.1186/s13630-016-0029-1 (PMC4827216; doi:10.1186/s13630-016-0029-1)

Additional file 5: Figure S3. Different classes of ciliary phenotypes. The ciliary localization of mRFP-ICK was studied in transiently transfected mIMCD3 cells. Five distinct expression patterns were classified; these are indicated above the upper images. Ciliary axonemes were visualized with anti-ARL13B (green), and ciliary transition zones that are present at the ciliary base were marked with anti-RPGRIP1L (pink). mRFP-ICK is shown in red.

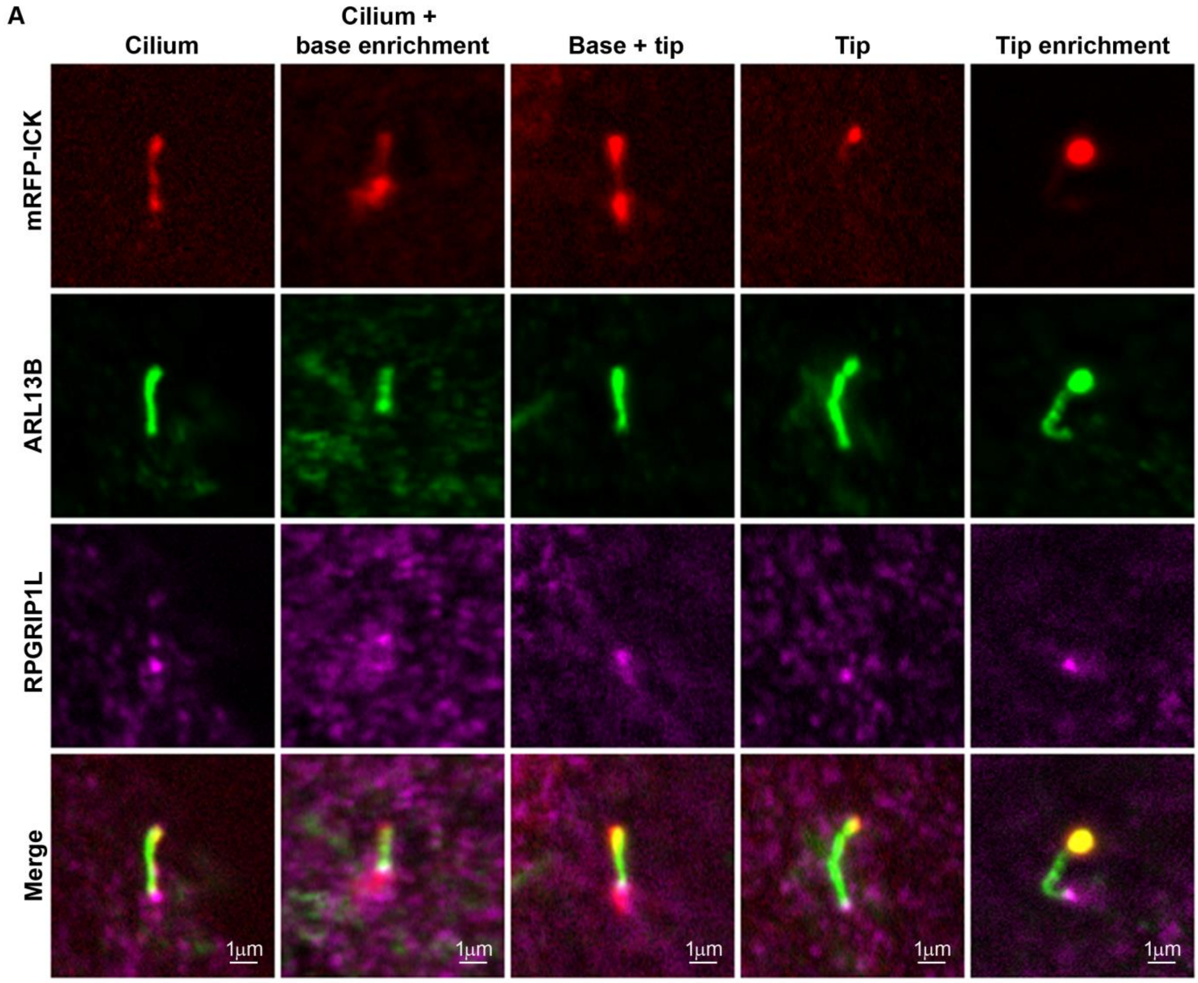

Supplement: Supplementary file 5 — 10.1186/s13630-016-0029-1 Different classes of ciliary phenotypes. [file 13630_2016_29_MOESM5_ESM.pdf]
