# Supplementary material for: A novel ICK mutation causes ciliary disruption and lethal endocrine-cerebro-osteodysplasia syndrome
Source: Cilia. 2016 Apr 11;5:8. doi: 10.1186/s13630-016-0029-1 (PMC4827216; doi:10.1186/s13630-016-0029-1)

Additional file 6: Figure S4. Ciliary length is not essentially altered in cilia of cells expressing mutant mRFP-ICK. IMCD3 cells were transiently transfected with wildtype or mutant (p.R272Q or p.G120C) mRFP-ICK and ciliary length was measured after 24 hours serum starvation to induce ciliogenesis. The length of cilia expressing mutant mRFP-ICK is not dramatically altered. The ciliary axoneme, visualized with ARL13B, of at least 28 cilia were measured per condition.

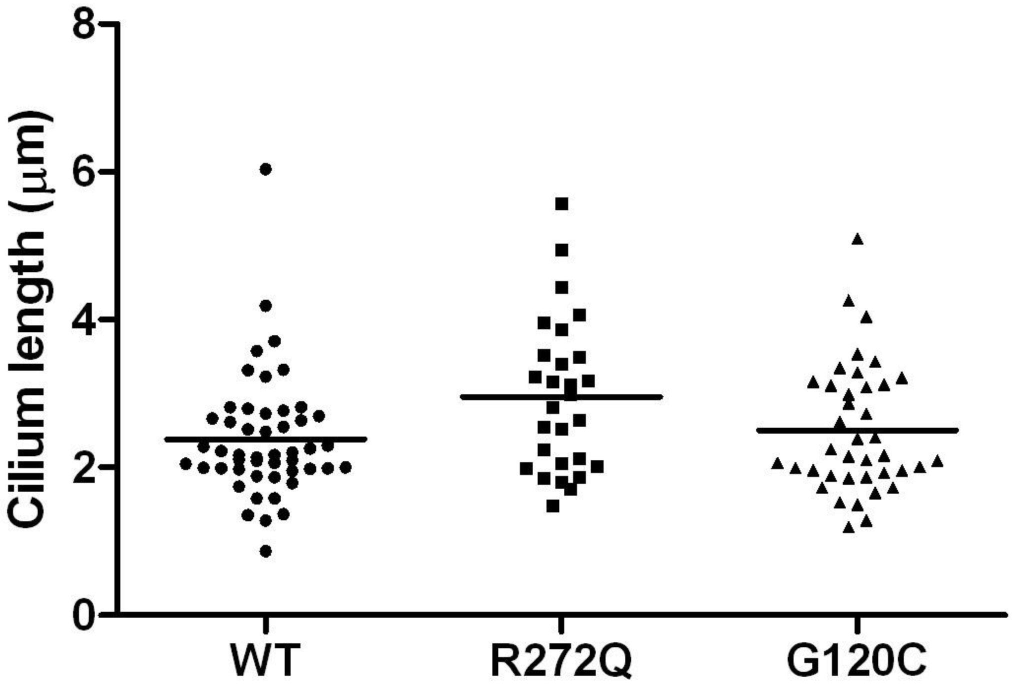

Supplement: Supplementary file 6 — 10.1186/s13630-016-0029-1 Ciliary length is not essentially altered in cilia of cells expressing mutant mRFP-ICK. [file 13630_2016_29_MOESM6_ESM.pdf]
