# Supplementary material for: A novel ICK mutation causes ciliary disruption and lethal endocrine-cerebro-osteodysplasia syndrome
Source: Cilia. 2016 Apr 11;5:8. doi: 10.1186/s13630-016-0029-1 (PMC4827216; doi:10.1186/s13630-016-0029-1)

Additional file 7: Figure S5. Ciliary length is not markedly altered in ECO patient-derived fibroblasts. The ciliary length of ECO patient-derived fibroblasts harboring a mutation in *ICK* at position c.815G>A; p.R272Q was compared to those of two healthy unrelated controls. Control I represents a non-Amish individual, while control II is from the Amish community. Natural variability in ciliary length is seen in control cell lines from different individuals as displayed in this graph; we can therefore only conclude that there are no major differences in the ciliary length of ECO patient-derived cells compared to control lines. Cells were serum starved for 48 hours to induce ciliogenesis and subsequently stained with ARL13B to visualize the ciliary axoneme. The ciliary axoneme of at least 125 cilia per cell line was measured.

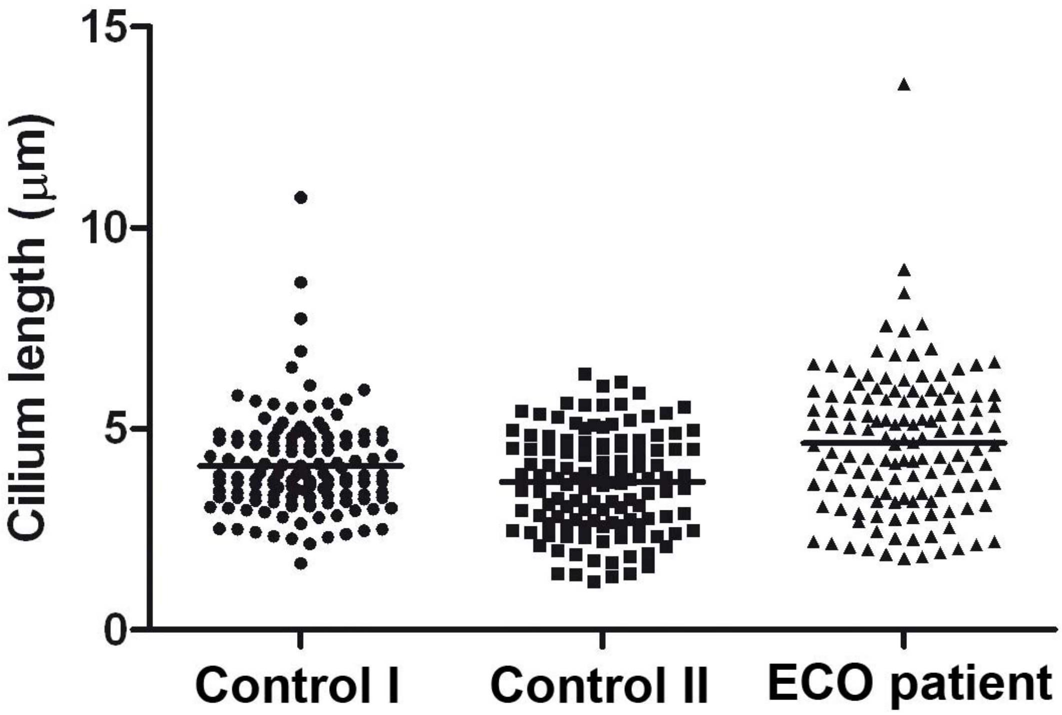

Supplement: Supplementary file 7 — 10.1186/s13630-016-0029-1 Ciliary length is not markedly altered in ECO patient-derived fibroblasts. [file 13630_2016_29_MOESM7_ESM.pdf]
